# Supplementary material for: Species distribution of Cannabis sativa: Past, present and future
Source: PLoS One. 2025 Mar 13;20(3):e0306007. doi: 10.1371/journal.pone.0306007 (PMC11906060; doi:10.1371/journal.pone.0306007)
Supplement: S1 Fig — 137 observations used for SDM model construction with a longitude greater than zero. Fig S2. Individual species distributions for each set of environmental properties examined (A) WorldClim Bioclimatic variables (B) ISRIC soil data (C) Solar radiation (kJm2/day) (D) Wind speed (m/s) (E) Water vapor pressure (kPa) (F) Elevation suitability maps. These maps were generated with Maxent using Worldclim and ISRIC data. Fig S3. Variable contribution graphs for each set of environmental properties examined (A) WorldClim Bioclimatic variables (B) ISRIC soil data (C) Solar radiation (kJm2/day) (D) Wind speed (m/s) (E) Water vapor pressure (kPa) and (F) Elevation. Fig S4. Area under the curve graphs each set of environmental properties examined (A) WorldClim Bioclimatic variables (B) ISRIC soil data (C) Solar radiation (kJm2/day) (D) Wind speed (m/s) (E) Water vapor pressure (kPa) and (F) Elevation. Fig S5. Overlay of all six environmental datasets (A) Worldwide plot (B) standard deviation for the overlay of all six environmental variables. These maps were generated with Maxent using Worldclim and ISRIC data. Fig S6. Overlay of all six environmental datasets (A) Variable contribution graph (B) Area under the curve graphs each set of environmental properties examined. Fig S7. Species distribution with temperature and precipitation data in Asia and Russia for (A) present day (B) SSP45 2050 (C) SSP45 2070 (D) SSP85 2050 (E) SSP85 2070. These maps were generated with Maxent using Worldclim data. Fig S8. Species distribution with temperature and precipitation data in Europe for (A) present day (B) SSP45 2050 (C) SSP45 2070 (D) SSP85 2050 (E) SSP85 2070. These maps were generated with Maxent using Worldclim data. Fig S9. Species distribution with temperature and precipitation data in the United States for (A) present day (B) SSP45 2050 (C) SSP45 2070 (D) SSP85 2050 (E) SSP85 2070. These maps were generated with Maxent using Worldclim data. Fig S10. Species distribution for a subset [file pone.0306007.s001.zip › S1 to 12 Fig/Supplemental_Figures_1-6.pdf]

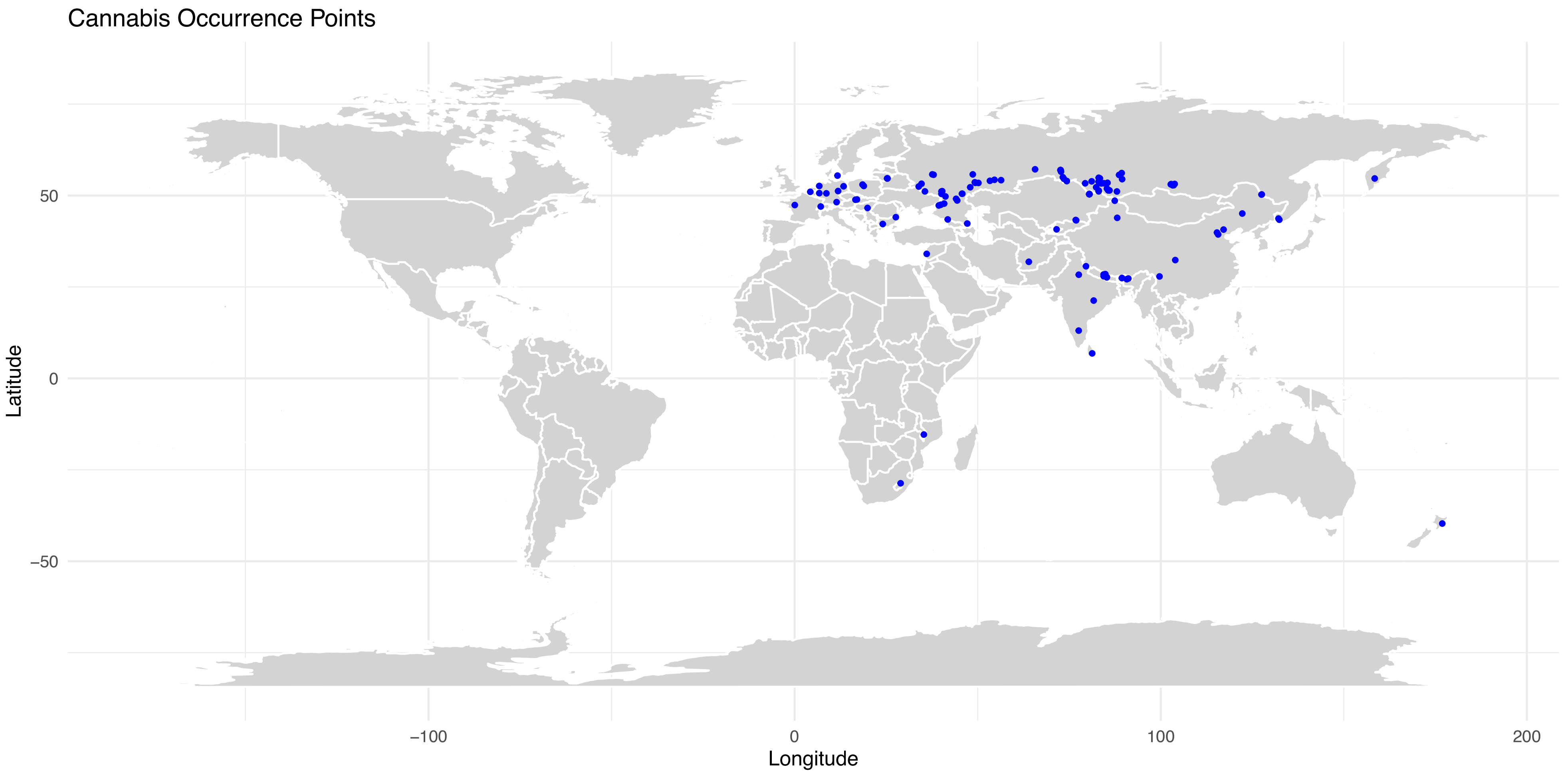

Supplemental Figure 1. 137 observations used for SDM model construction with a longitude greater than zero.

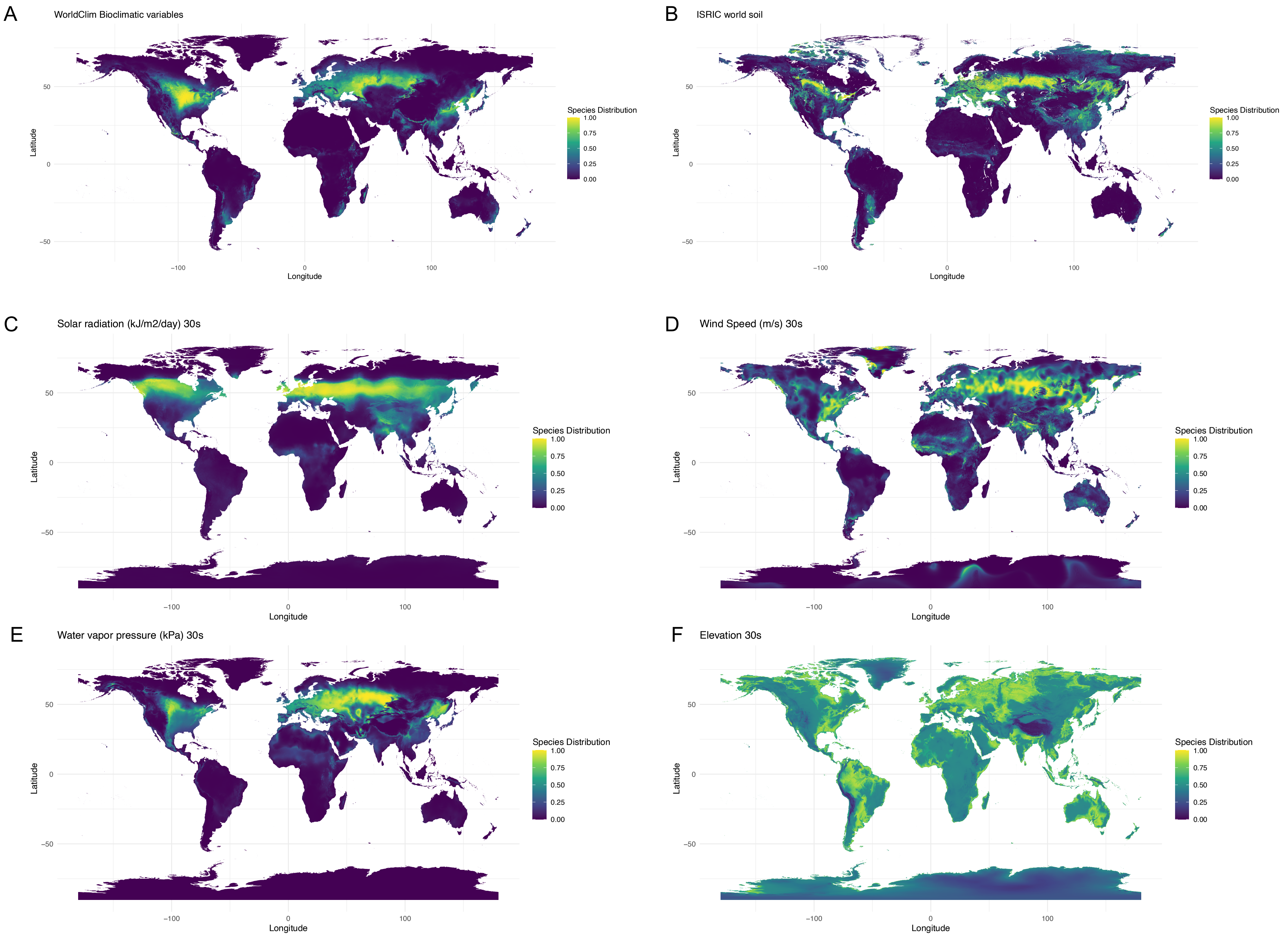

Supplemental Figure 2. Individual species distributions for each set of environmental properties examined **(A)** WorldClim Bioclimatic variables **(B)** ISRIC soil data **(C)** Solar radiation (kJm^2/day) **(D)** Wind speed (m/s) **(E)** Water vapor pressure (kPa) **(F)** Elevation suitability maps.

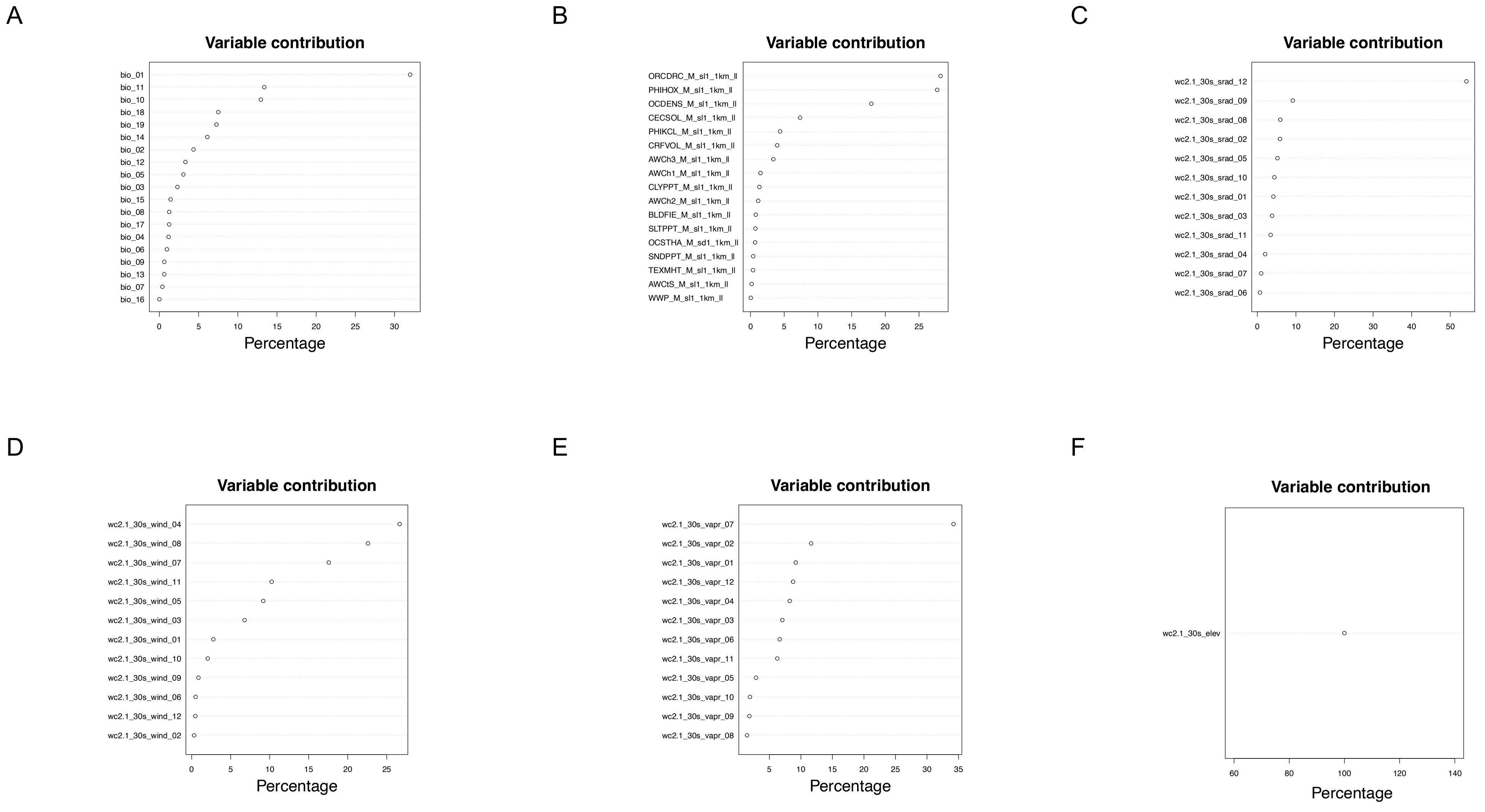

Supplemental Figure 3. Variable contribution graphs for each set of environmental properties examined **(A)** WorldClim Bioclimatic variables **(B)** ISRIC soil data **(C)** Solar radiation (kJm^2/day) **(D)** Wind speed (m/s) **(E)** Water vapor pressure (kPa) and **(F)** Elevation.

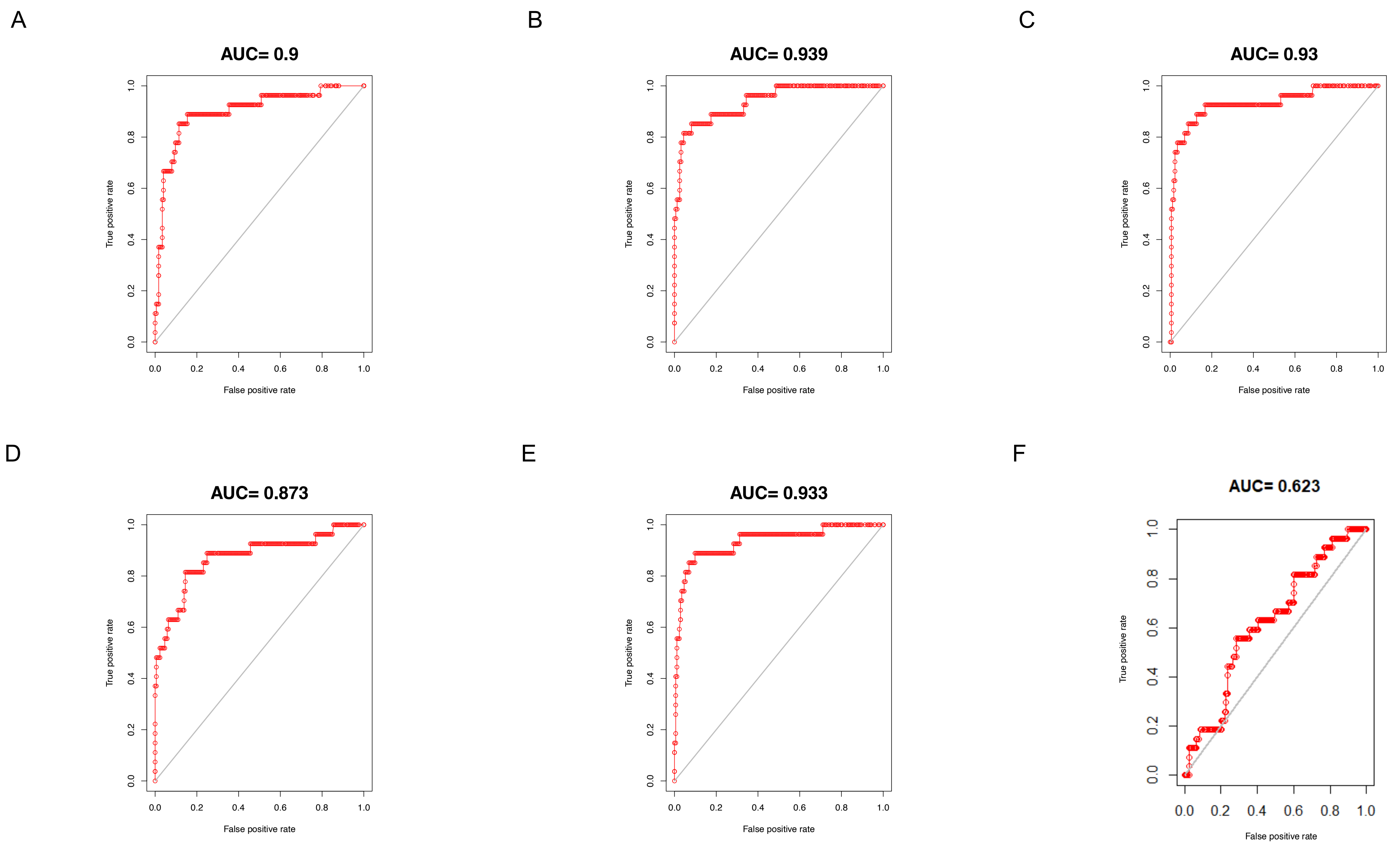

Supplemental Figure 4. Area under the curve graphs each set of environmental properties examined **(A)** WorldClim Bioclimatic variables **(B)** ISRIC soil data **(C)** Solar radiation (kJm^2/day) **(D)** Wind speed (m/s) **(E)** Water vapor pressure (kPa) and **(F)** Elevation.

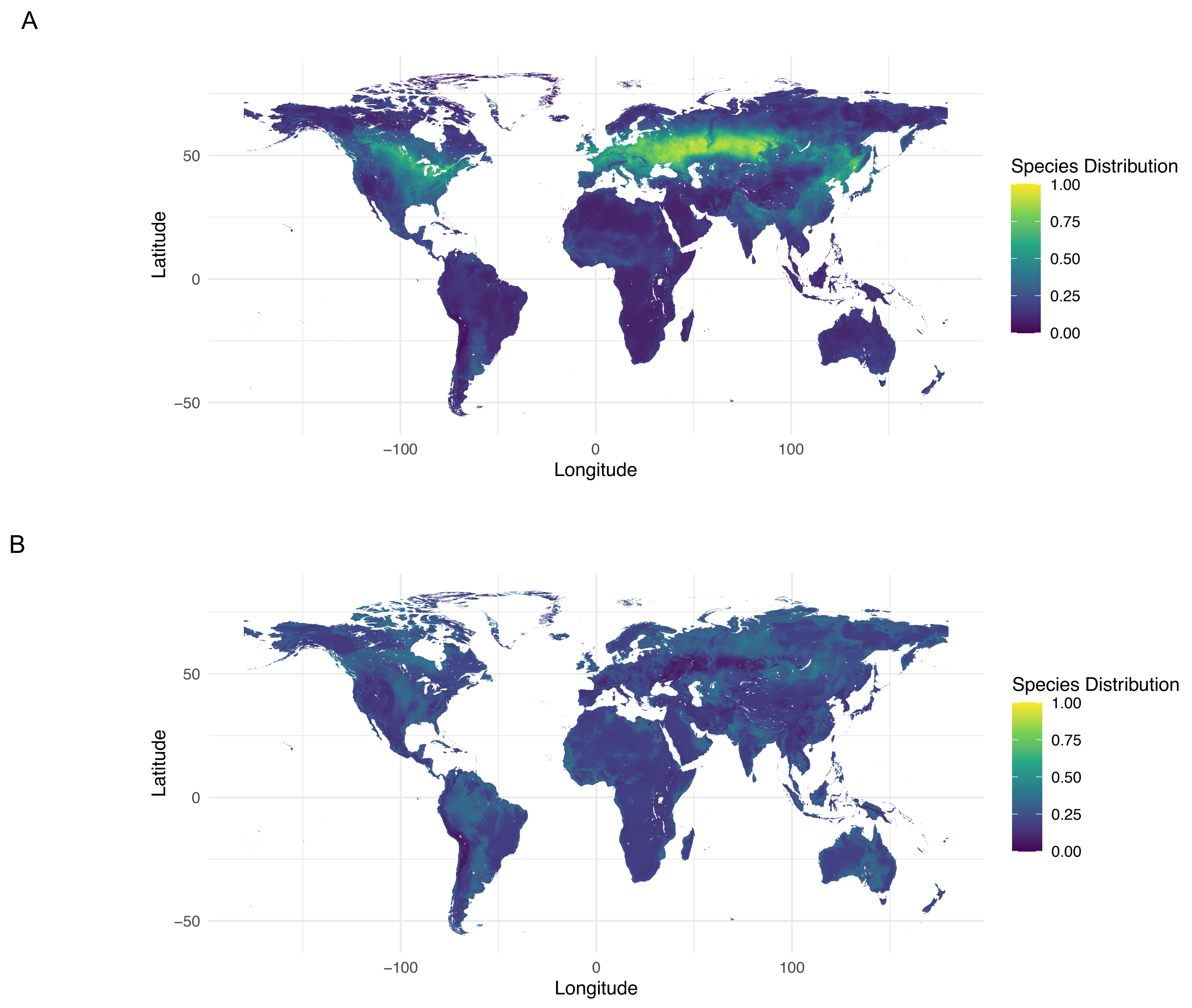

Supplemental Figure 5. Overlay of all six environmental datasets **(A)** Worldwide plot **(B)** standard deviation for the overlay of all six environmental variables.

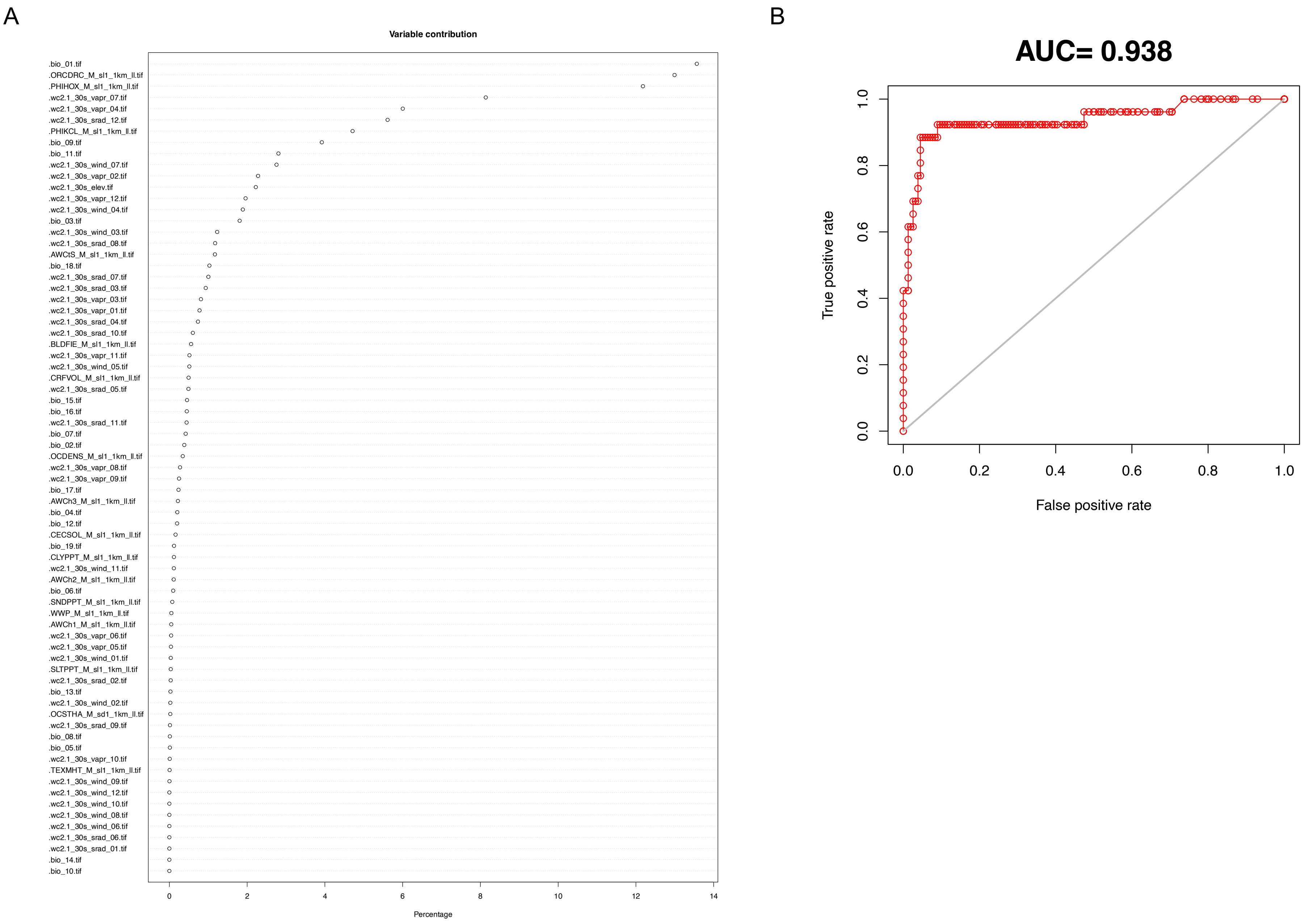

Supplemental Figure 6. Overlay of all six environmental datasets **(A)** Variable contribution graph (B) Area under the curve graphs each set of environmental properties examined
