# Supplementary material for: Species distribution of Cannabis sativa: Past, present and future
Source: PLoS One. 2025 Mar 13;20(3):e0306007. doi: 10.1371/journal.pone.0306007 (PMC11906060; doi:10.1371/journal.pone.0306007)
Supplement: S1 Fig — 137 observations used for SDM model construction with a longitude greater than zero. Fig S2. Individual species distributions for each set of environmental properties examined (A) WorldClim Bioclimatic variables (B) ISRIC soil data (C) Solar radiation (kJm2/day) (D) Wind speed (m/s) (E) Water vapor pressure (kPa) (F) Elevation suitability maps. These maps were generated with Maxent using Worldclim and ISRIC data. Fig S3. Variable contribution graphs for each set of environmental properties examined (A) WorldClim Bioclimatic variables (B) ISRIC soil data (C) Solar radiation (kJm2/day) (D) Wind speed (m/s) (E) Water vapor pressure (kPa) and (F) Elevation. Fig S4. Area under the curve graphs each set of environmental properties examined (A) WorldClim Bioclimatic variables (B) ISRIC soil data (C) Solar radiation (kJm2/day) (D) Wind speed (m/s) (E) Water vapor pressure (kPa) and (F) Elevation. Fig S5. Overlay of all six environmental datasets (A) Worldwide plot (B) standard deviation for the overlay of all six environmental variables. These maps were generated with Maxent using Worldclim and ISRIC data. Fig S6. Overlay of all six environmental datasets (A) Variable contribution graph (B) Area under the curve graphs each set of environmental properties examined. Fig S7. Species distribution with temperature and precipitation data in Asia and Russia for (A) present day (B) SSP45 2050 (C) SSP45 2070 (D) SSP85 2050 (E) SSP85 2070. These maps were generated with Maxent using Worldclim data. Fig S8. Species distribution with temperature and precipitation data in Europe for (A) present day (B) SSP45 2050 (C) SSP45 2070 (D) SSP85 2050 (E) SSP85 2070. These maps were generated with Maxent using Worldclim data. Fig S9. Species distribution with temperature and precipitation data in the United States for (A) present day (B) SSP45 2050 (C) SSP45 2070 (D) SSP85 2050 (E) SSP85 2070. These maps were generated with Maxent using Worldclim data. Fig S10. Species distribution for a subset [file pone.0306007.s001.zip › S1 to 12 Fig/Supplemental_Figures_7-12.pdf]

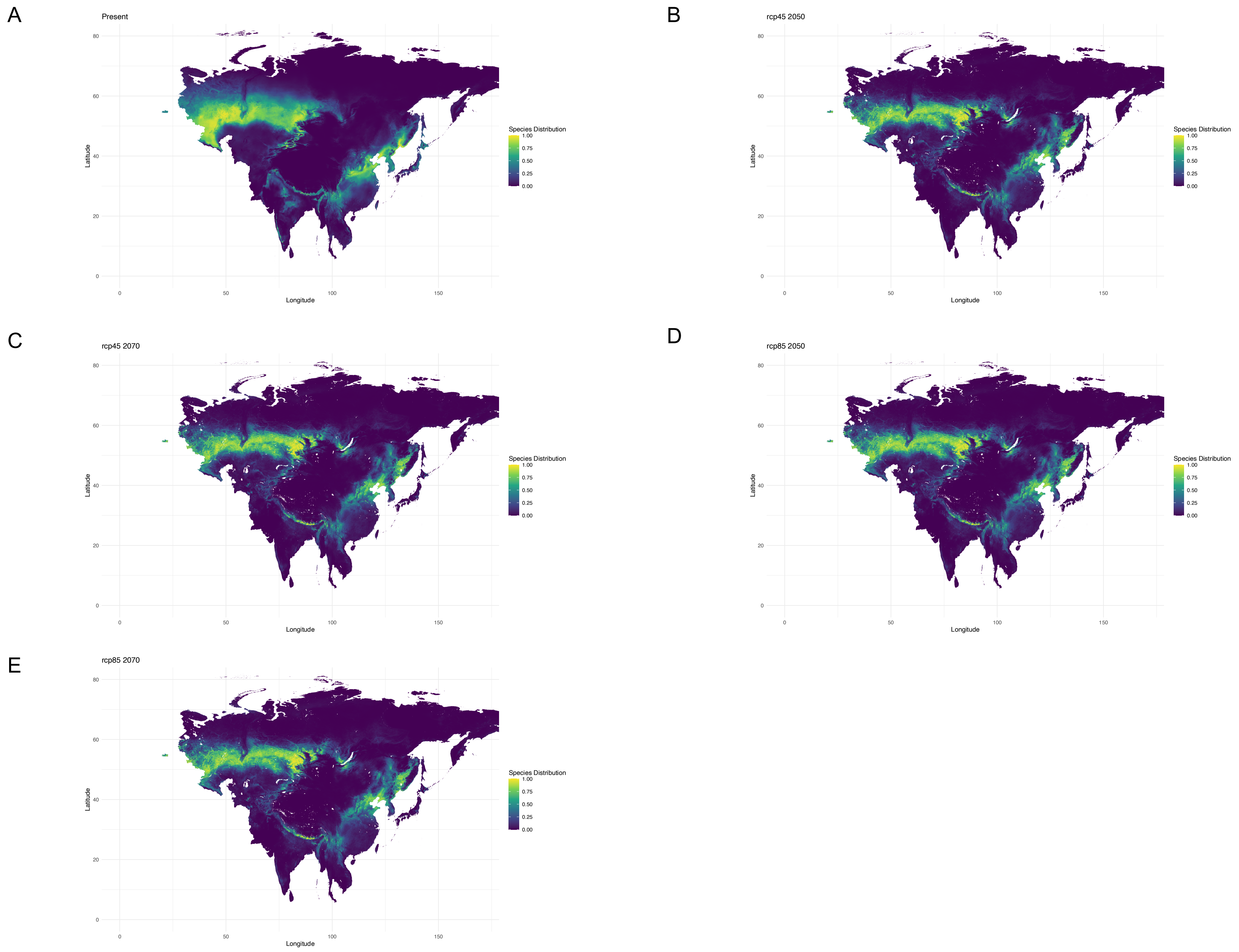

Supplemental Figure 7. Species distribution with temperature and precipitation data in Asia and Russia for **(A)** present day **(B)** SSP45 2050 **(C)** SSP45 2070 **(D)** SSP85 2050 **(E)** SSP85 2070.

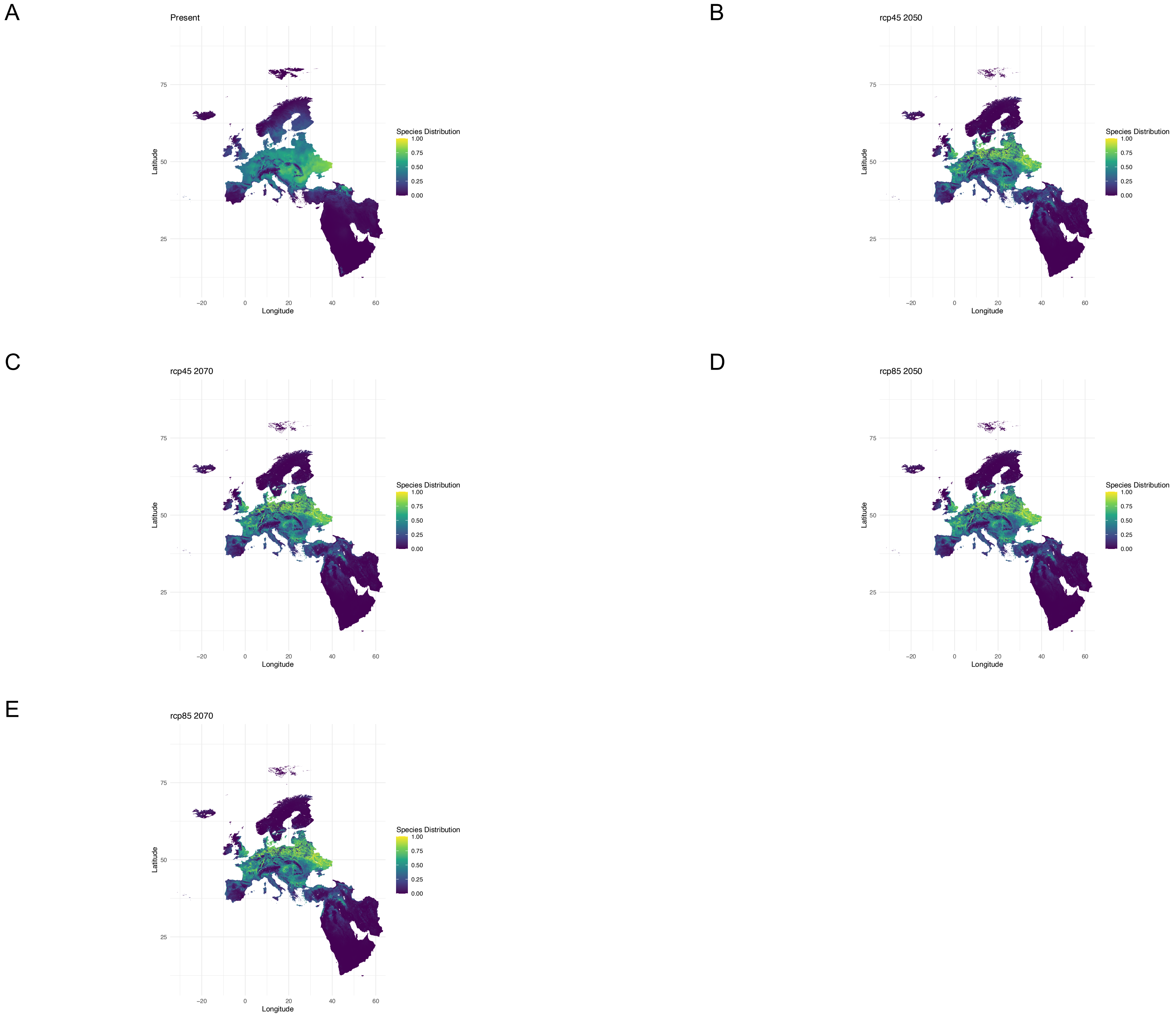

Supplemental Figure 8. Species distribution with temperature and precipitation data in Europe for **(A)** present day **(B)** SSP45 2050 **(C)** SSP45 2070 **(D)** SSP85 2050 **(E)** SSP85 2070.

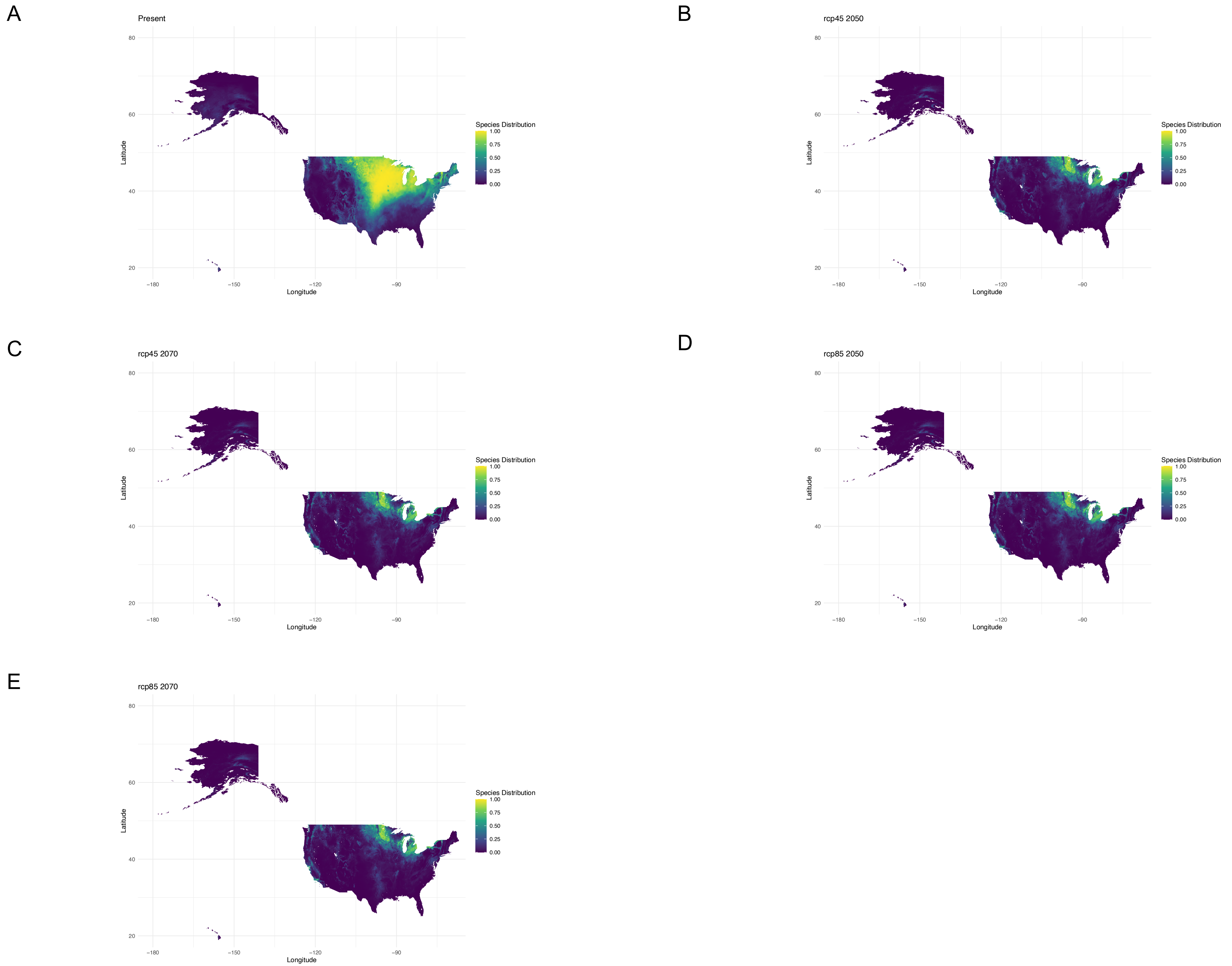

Supplemental Figure 9. Species distribution with temperature and precipitation data in the United States for **(A)** present day **(B)** SSP45 2050 **(C)** SSP45 2070 **(D)** SSP85 2050 **(E)** SSP85 2070.

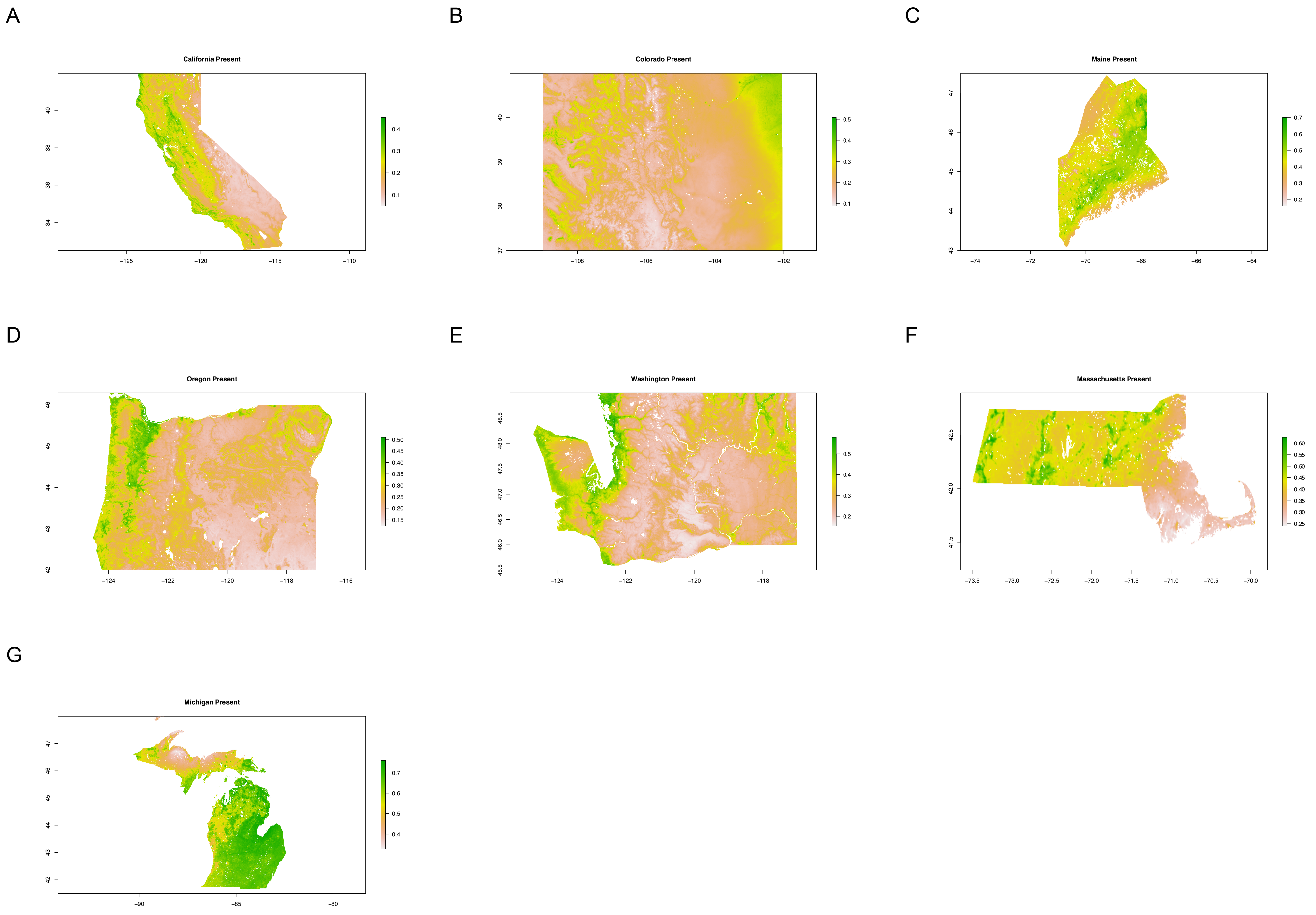

Supplemental Figure 10. Species distribution for a subset of the United States with data for all six environmental properties examined **(A)** California **(B)** Colorado **(C)** Maine **(D)** Oregon **(E)** Washington **(F)** Massachusetts **(G)** Michigan.

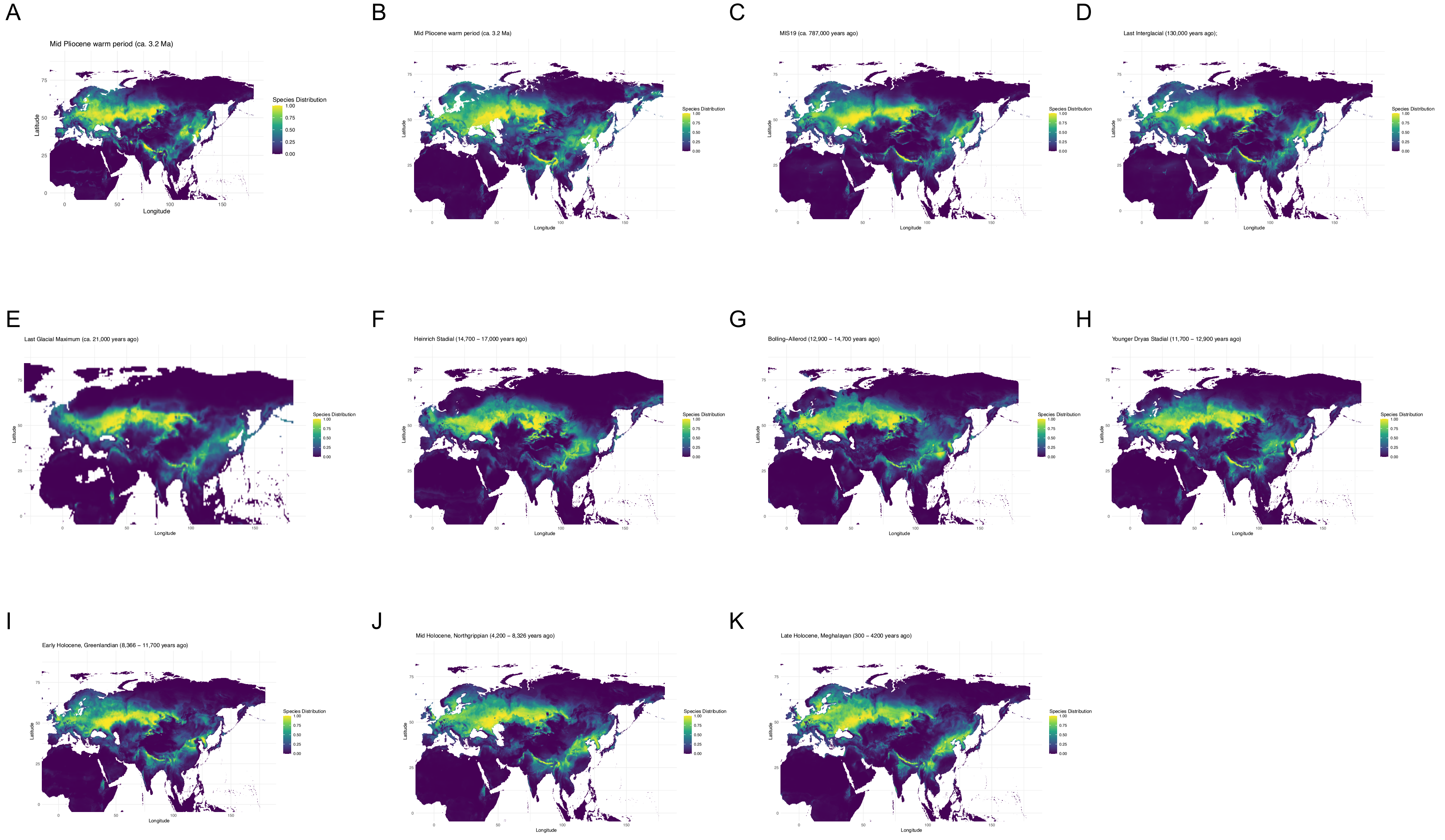

Supplemental Figure 11. (A) Pleistocene: M2 (ca. 3.3 Ma) **(B)** Predicted Distribution for the Paleoclimate timepoint of the Mid Pliocene warm period (ca. 3.2 Ma) **(C)** Predicted Distribution for the Paleoclimate timepoint of the Pleistocene: MIS19 (ca. 787,000 years ago) **(D)** Predicted Distribution for the Paleoclimate timepoint of the Pleistocene: Last Interglacial (130,000 years ago) **(E)** Predicted Distribution for the Paleoclimate timepoint of the Pleistocene: Last Glacial Maximum (ca. 21,000 years ago) **(F)** Potential Distribution for the Paleoclimate timepoint of the Pleistocene: Heinrich Stadial (14,700 – 17,000 years ago) **(G)** Potential Distribution for the Paleoclimate timepoint of the Pleistocene: Bolling-Allerod (12,900 – 14,700 years ago) **(H)** Potential Distribution for the Paleoclimate timepoint of the Pleistocene: Younger Dryas Stadial (11,700 – 12,900 years ago) **(I)** Potential Distribution for the Paleoclimate timepoint of the Pleistocene: Early Holocene, Greenlandian (8,366 - 11,700 years ago) **(J)** Potential Distribution for the Paleoclimate timepoint of the Pleistocene: Mid Holocene, Northgrippian (4,200 – 8,326 years ago) **(K)** Potential Distribution for the Paleoclimate timepoint of the Pleistocene: Late Holocene, Meghalayan (300 – 4200 years ago)

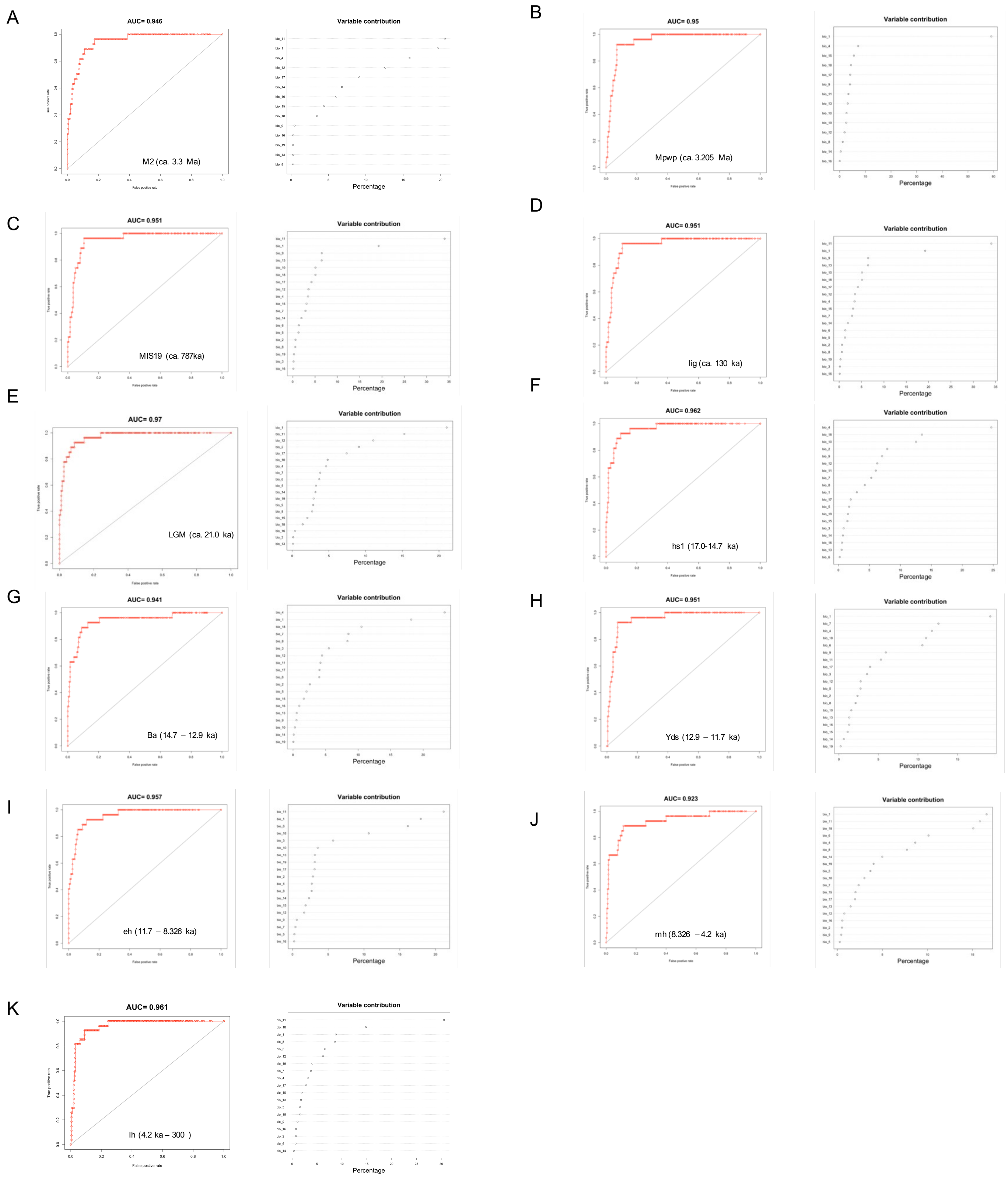

Supplemental Figure 12. AUC and variable contribution graphs for each timepoint from the Paleoclim dataset **(A)** Pleistocene: M2 (ca. 3.3 Ma) **(B)** Mid Pliocene warm period (ca. 3.2 Ma) **(C)** Pleistocene: MIS19 (ca. 787,000 years ago) **(D)** Pleistocene: Last Interglacial (130,000 years ago) **(E)** Last Glacial Maximum (ca. 21,000 years ago) **(F)** Heinrich Stadial (14,700 – 17,000 years ago **(G)** Bolling-Allerod (12,900 – 14,700 years ago) **(H)** Younger Dryas Stadial (11,700 – 12,900 years ago) **(I)** Early Holocene, Greenlandian (11,700-8,326 years ago) **(J)** Mid Holocene, Northgrippian (4,200 – 8,326 years ago) **(K)** Late Holocene, Meghalayan (300 – 4200 years ago)
