# Supplementary material for: Species distribution of Cannabis sativa: Past, present and future
Source: PLoS One. 2025 Mar 13;20(3):e0306007. doi: 10.1371/journal.pone.0306007 (PMC11906060; doi:10.1371/journal.pone.0306007)
Supplement: Attachment — Submitted filename: Cannabis Distribution Response to Reviewer Comments PlosOne.docx [file pone.0306007.s004.docx]

**Response To reviewer Comments**

**Editor:**

Thank you for the opportunity to revise the manuscript. The comments were very helpful and we feel they have greatly improved the manuscript.

**Comment 1:** When submitting your revision, we need you to address these additional requirements. Please ensure that your manuscript meets PLOS ONE's style requirements, including those for file naming. The PLOS ONE style templates can be found at
[https://journals.plos.org/plosone/s/file?id=wjVg/PLOSOne_formatting_sample_main_body.pdf](https://urldefense.com/v3/__https://journals.plos.org/plosone/s/file?id=wjVg*PLOSOne_formatting_sample_main_body.pdf__;Lw!!PvDODwlR4mBZyAb0!TmvrSpMrmag0US99eibccY7kk-9S-G5EZRn3mqWWEgZ4kZfM1Z5XhAogGnvxC7q_00PiPpNkVHdeOckSVg$) and
[https://journals.plos.org/plosone/s/file?id=ba62/PLOSOne_formatting_sample_title_authors_affiliations.pdf](https://urldefense.com/v3/__https://journals.plos.org/plosone/s/file?id=ba62*PLOSOne_formatting_sample_title_authors_affiliations.pdf__;Lw!!PvDODwlR4mBZyAb0!TmvrSpMrmag0US99eibccY7kk-9S-G5EZRn3mqWWEgZ4kZfM1Z5XhAogGnvxC7q_00PiPpNkVHcbC5mIJg$)

**Response:** We have modified the manuscript to fit the template and the reference scheme changed to numbers

**Comment 2:** Thank you for stating the following in the Competing Interests section: "LeafWorks Inc. is a for profit company." We note that you received funding from a commercial source: LeafWorks Inc. Please provide an amended Competing Interests Statement that explicitly states this commercial funder, along with any other relevant declarations relating to employment, consultancy, patents, products in development, marketed products, etc. Within this Competing Interests Statement, please confirm that this does not alter your adherence to all PLOS ONE policies on sharing data and materials by including the following statement: ""This does not alter our adherence to PLOS ONE policies on sharing data and materials.” (as detailed online in our guide for authors [http://journals.plos.org/plosone/s/competing-interests).](https://urldefense.com/v3/__http://journals.plos.org/plosone/s/competing-interests).__;!!PvDODwlR4mBZyAb0!TmvrSpMrmag0US99eibccY7kk-9S-G5EZRn3mqWWEgZ4kZfM1Z5XhAogGnvxC7q_00PiPpNkVHdep_SOQA$)  If there are restrictions on sharing of data and/or materials, please state these. Please note that we cannot proceed with consideration of your article until this information has been declared. Please include your amended Competing Interests Statement within your cover letter. We will change the online submission form on your behalf.

**Response:** We have changed the Conflict of Interest Statement to

“LeafWorks is a for profit company that provided funding to help with the computing for this manuscript. This does not alter our adherence to PLOS ONE policies on sharing data and materials.”

**Comment 3:** We note that Figures 1,2,3,4, S1,S2,S5,S7,S8,S9,S10 and S11 in your submission contain [map/satellite] images which may be copyrighted.

USGS National Map Viewer (public domain): [http://viewer.nationalmap.gov/viewer/](https://urldefense.com/v3/__http://viewer.nationalmap.gov/viewer/__;!!PvDODwlR4mBZyAb0!TmvrSpMrmag0US99eibccY7kk-9S-G5EZRn3mqWWEgZ4kZfM1Z5XhAogGnvxC7q_00PiPpNkVHe1GCX9iA$)
The Gateway to Astronaut Photography of Earth (public domain): [http://eol.jsc.nasa.gov/sseop/clickmap/](https://urldefense.com/v3/__http://eol.jsc.nasa.gov/sseop/clickmap/__;!!PvDODwlR4mBZyAb0!TmvrSpMrmag0US99eibccY7kk-9S-G5EZRn3mqWWEgZ4kZfM1Z5XhAogGnvxC7q_00PiPpNkVHdFdJRs7g$)
Maps at the CIA (public domain): [https://www.cia.gov/library/publications/the-world-factbook/index.html](https://urldefense.com/v3/__https://www.cia.gov/library/publications/the-world-factbook/index.html__;!!PvDODwlR4mBZyAb0!TmvrSpMrmag0US99eibccY7kk-9S-G5EZRn3mqWWEgZ4kZfM1Z5XhAogGnvxC7q_00PiPpNkVHfxKrZdXw$) and [https://www.cia.gov/library/publications/cia-maps-publications/index.html](https://urldefense.com/v3/__https://www.cia.gov/library/publications/cia-maps-publications/index.html__;!!PvDODwlR4mBZyAb0!TmvrSpMrmag0US99eibccY7kk-9S-G5EZRn3mqWWEgZ4kZfM1Z5XhAogGnvxC7q_00PiPpNkVHf1qIQpGw$)
NASA Earth Observatory (public domain): [http://earthobservatory.nasa.gov/](https://urldefense.com/v3/__http://earthobservatory.nasa.gov/__;!!PvDODwlR4mBZyAb0!TmvrSpMrmag0US99eibccY7kk-9S-G5EZRn3mqWWEgZ4kZfM1Z5XhAogGnvxC7q_00PiPpNkVHcs7pc4Lw$)
Landsat: [http://landsat.visibleearth.nasa.gov/](https://urldefense.com/v3/__http://landsat.visibleearth.nasa.gov/__;!!PvDODwlR4mBZyAb0!TmvrSpMrmag0US99eibccY7kk-9S-G5EZRn3mqWWEgZ4kZfM1Z5XhAogGnvxC7q_00PiPpNkVHdYJBgDWw$)
USGS EROS (Earth Resources Observatory and Science (EROS) Center) (public domain): [http://eros.usgs.gov/#](https://urldefense.com/v3/__http://eros.usgs.gov/*__;Iw!!PvDODwlR4mBZyAb0!TmvrSpMrmag0US99eibccY7kk-9S-G5EZRn3mqWWEgZ4kZfM1Z5XhAogGnvxC7q_00PiPpNkVHcmqPd1zQ$)
Natural Earth (public domain): [http://www.naturalearthdata.com/](https://urldefense.com/v3/__http://www.naturalearthdata.com/__;!!PvDODwlR4mBZyAb0!TmvrSpMrmag0US99eibccY7kk-9S-G5EZRn3mqWWEgZ4kZfM1Z5XhAogGnvxC7q_00PiPpNkVHd6MAweuA$)

**Response:** These images do not actually contain satellite data, the input rasters are from worldclim and ISRIC and the images and colouring generated is based on the analysis done in this manuscript using Maxent. The viridis palette was used in the final plots.

**Comment 4:**  Please include captions for your Supporting Information files at the end of your manuscript, and update any in-text citations to match accordingly. Please see our Supporting Information guidelines for more information: [http://journals.plos.org/plosone/s/supporting-information](https://urldefense.com/v3/__http://journals.plos.org/plosone/s/supporting-information__;!!PvDODwlR4mBZyAb0!TmvrSpMrmag0US99eibccY7kk-9S-G5EZRn3mqWWEgZ4kZfM1Z5XhAogGnvxC7q_00PiPpNkVHebL11ulQ$).

**Response:** We have added the captions.

**Reviewer #1 comments:**

**Comment 1:** The article attempts to depict the past, present, and future distribution of hemp species using available global data. Although the paper is well-structured, I found the results and discussion sections difficult to follow due to the numerous figures and tables. I suggest adding a separate section focused directly on the discussion. The methodology is straightforward, but there is a significant gap in training data from other continents, which the authors did not address or discuss the implications of. While the authors used freely available global data, the accuracy of this data, especially soil information, is questionable and should be properly discussed in the paper.

**Response:** Thank you for this summary. We have added cavates regarding the soil data. The below has been added to the end of the suitability and soil section in the results & discussion section for this purpose.

*“There are a number of limitations and biases associated with the climate and soils data that need to be considered. The limitations include the resolution of the bioclimatic and biophysical data and the assumptions made in the interpolation of these data. This is particularly true for biophysical (soil) variables, which likely vary over finer scales than the database which impact the interpolation techniques (Brady et al. 2005). “*

Brady, K. U., A. R. Kruckeberg, and H. D. Bradshaw, Jr., 2005 Evolutionary ecology of plant adaptation to serpentine soils. Annu. Rev. Ecol. Evol. Syst. 36: 243–266.

As regards the significant gap in training data from other continents - as we are dealing with suspected wild plants, we wouldn't expect to use data points from other continents for training as the native range for cannabis is Eurasia. We have added the below into the text to elaborate on why eurasian data points were selected in the materials and methods

*“After filtering for a longitude greater than zero, 137 observations remained and were used for SDM model construction (****Table S3; Fig. S1****). Eurasia is considered the center of origin of Cannabis, which provided rationale for including only data points with a longitude greater than zero in this study.”*

**Comment 2:**  "Cannabis now considered a monotypic genus". This is still a matter of debate, particularly when we consider Cannabis ruderalis. Please add more context here.
**Response:** Cannabis ruderalis could be considered in some more northern Russian latitudes where it grows freely, a wild relative. These plants have been used widely for the introgression of day neutrality in flowering time. Due to this fertility between all cannabis types when crossed (hemp, ruderal, recreational) it is clear there is no species barrier. There is considerable debate about if species delineations are therefore appropriate given this cross variety fertility. Only one paper to date (Ren et al, 2021) includes a genetic comparison of wild or feral, hemp type and THC-dominant type cannabis samples - and while the feral sampled varieties are most basal in the phylogeny, this genetic distance does not seem to affect crossing compatibility. The below has been included in the introduction to add more nuance

*“Historically, it has been broadly divided into two types, hemp- or drug-type, with early descriptions dating back to Linnaeus (1753) and Lamarck (1785) (Linnaeus, 1753; Lamark, 1783). Linnaeus described the plants from Northern Europe as Cannabis sativa and Lamarck described plants from India as Cannabis indica. In 1924 an additional purported wild species growing in central Russia was described by Janischewsky (Janischewsky, 1924) and termed Cannabis ruderalis. At the time, morphological differences between these three taxa led to the proposition of multiple species (sativa, indica and ruderalis) however, more recent work supports the rank of subspecies (McParland, 2018) with genetic diversity occurring across a latitudinal gradient along which classic differentiating phenotypes occur (Zhang et al., 2018). Due to the cross fertility of the proposed multispecies, Cannabis is now more commonly considered a monotypic genus (Small, 2017; Barcaccia et al., 2020; Kocalchuck et al., 2020)”*

**Comment 3:**  "Identifying the best climatic and soil conditions for growth informs nutrient and water management and approaches such as species distribution modeling (SDM) providing a means for more informed land selection that may facilitate minimizing negative environmental externalities (Mehrabi et al., 2019)"

This sentence contains many ideas and the reference seems to be for sunflower. Please re-write it and provide a more relavant reference.

**Response:** We have rewritten the sentence

*“Under climate change it is very possible that in the future different regions may be more appropriate for different crops (Pironon et al., 2019). Species distribution modeling (SDM) provides a method for informed land selection by identifying regions with favorable climatic and soil conditions and providing a suitability score for identifying these regions.”*

*Pironon, S., Etherington, T. R., Borrell, J. S., Kühn, N., Macias-Fauria, M., Ondo, I., et al. (2019). Potential adaptive strategies for 29 sub-Saharan crops under future climate change. Nature Climate Change, 9(10), 758-763.*

**Comment 4:** Instead of just pasting the link, provide a proper citation for the Economist article about Cannabis market in California.
**Response:** We have changed this reference to (Walsh, 2023). Full reference is: Walsh, Dustin. "Michigan's weed market is now the top in the nation." *Crain's Detroit Business*, vol. 39, no. 33, 28 Aug. 2023, p. 0005. *Gale OneFile: Health and Medicine*, link.gale.com/apps/doc/A762882796/HRCA?u=albe12389&sid=googleScholar&xid=f17ee90f.”

No issue number could be found for the economist article or author, only the date it was published online: May 14th 2022 (<https://www.economist.com/united-states/2022/05/14/in-california-the-worlds-largest-legal-weed-market-is-going-up-in-smoke>**)**

**Comment 5:** Whilst the introduction section is well organised, it lacks depth and proper linkage to the 'knowledge gap' the article is trying to fill. More importantly, the focus of the article seems to be a study of status and shift of cultivation at the global level. Bud the authors suddenly add information about the cultivation center in California. I suggest the authors expand the introduction section and provide a better linkage to the aim and objectives of the study.
**Response:** The knowledge gap and rationale for this study was that no prior species distribution modeling for cannabis has been published for present day or future climate scenarios. We aimed to provide a quantitative analysis on how favorable ranges of Cannabis may change moving towards the various future climate scenarios. The introduction has been amended to include the below to better link the aim and objectives of the study and why the focus was directed to California more specifically

*“Despite this widespread cultivation, no prior species distribution modeling for Cannabis has been published for present day or future scenarios”*

*“Species distribution modeling (SDM) provides a method for informed land selection by identifying regions with favorable climatic and soil conditions and providing a suitability score for identifying these regions. SDM can also support conservation decision making by identifying where other suitable habitats may be for endangered species and identifying the rank in important environmental factors involved in their distribution.”*

*“However it is currently unknown how future climate change may impact Cannabis cultivation and conservation globally and what are the most important environmental variables that explain Cannabis species distribution.”*

**Comment 6:**  Please provide a proper citation for the GBIF occurrence data.
**Response:** GBIF suggests this is how data to be cited: GBIF.org (20 April 2020) GBIF Occurrence Download <https://doi.org/10.15468/dl.d8n6hx> and this is what is referenced in the document. Please see below image for rationale.

**Comment 7:** Were there other occurrence data for C. Sative on GBIF?
**Response:** There are many other occurrence data for C.sativa on GBIF, however only data points which had associated images were included in this analysis, reducing the total number of GBIF records dramatically. Without being able to see the plants surroundings, it would be impossible to know if the plant was growing wild and without human intervention and so occurrence points with no associated image were not included.

**Comment 8:** "Of these, 302 were deemed as wild or escapees and growing without human intervention " how they were deemed wild? what was the method?
**Response:** For the data points used in the study, associated images for each latitude and longitude were individually examined and the data point was only included where it was clear the plant was growing wild and most likely without any human intervention. The call on wild was made if no man made objects of any kind were seen in the photo and that the plant appeared to be growing along with others or in a landscape that appeared to not be being managed. In table S1 the associated links to the images for each datapoint that was assessed are included. In this version of the tables, the link to the corresponding images is included for the 137 datapoints (Table S3) used in the downstream analyses.

The following has been added to the materials and methods “*Plants were classified as wild if image inspection revealed no visible man-made objects of any kind, the plant was growing amongst other plants and the landscape appeared unmanaged*.”

**Comment 9:** Table Table S3 137 in Supplementary Materials is empty!
**Response:** Apologies these data points have been included in this version of the tables.

**Comment 10:** The ISRIC soil data were old. Can you use OpenLandMap or [https://soil.copernicus.org/articles/7/217/2021/?](https://urldefense.com/v3/__https://soil.copernicus.org/articles/7/217/2021/?__;!!PvDODwlR4mBZyAb0!TmvrSpMrmag0US99eibccY7kk-9S-G5EZRn3mqWWEgZ4kZfM1Z5XhAogGnvxC7q_00PiPpNkVHcIZLzF2Q$)
**Response:** We thank the reviewer for pointing this out. We are not able to redo the analysis with this data, but we have added caveats about the data being used. The following has been added to the text of the discussion.

*“There are a number of limitations and biases associated with the climate and soils data that need to be considered. The limitations include the resolution of the bioclimatic and biophysical data and the assumptions made in the interpolation of these data. This is particularly true for biophysical (soil) variables, which likely vary over finer scales than the database which impact the interpolation techniques (Brady et al. 2005).*”

**Comment 11:** Please create proper citations for URLs in the methodology section.
**Response:** The links to worldclim and isric have been removed leaving just the primary references for these. A citation was possible for the EarthWorks shapefiles, but none was found for the world administrative boundaries shape file, however the link was removed as requested. Remaining links are for the source code github and high resolution figures.

**Comment 12:** Why different sources was used for admin boundaries shapefiles? Was GADM database not comprehensive enough?
**Response:** There was no specific reason for using different shapefiles other than prior knowledge of the world administrative boundaries and earthworks Standford sources.

**Comment 13:** Please add a better description of how historical SDMs were created. How they were validated?

**Response:** Historical SDM were created similarly to the present day SDM using Maxent. Present day latitude and longitude datapoints (n=137) were used under the assumption that where cannabis grows wild today may reflect where it could have grown in the past. The Paleoclimate was instead the input raster for the conditions at each time point. Without samples with geo-references and pollen dating, it is not possible to validate the historical SDM. This is why they are a supplemental figure. It is acknowledged that assumptions were made about present day and past ranges to conduct this analysis.

The following has been added to the text of the manuscript:

“*Paleoclimate data were sourced from paleoclim.org with the highest spatial resolution of 2.5 arc-minutes (~5km) downloaded (****Table S6,*** *(Fordham et al., 2917)) with suitability maps were created using the Maxent software (Version 3.3.4). For historic distribution models, present day latitude and longitude data points (n=137) were used under the assumption that where cannabis grows wild today may reflect where it could have grown in the past.*”

As well as the following to results and discussion section:

*“Archeological evidence, including carbon dated pollen samples across the species distribution range modeled here is still needed to support and validate the findings presented in this study.*”

**Comment 14:** Although both "suitability" and "species occurrrence" have been used interchangeably, suitability encompasses a much bigger area and in this context, I advice authors to avoid use of the word 'suitablity', particularly when they are referring to the past coditions.
**Response:** Suitability in this instance describes an area where environmental conditions at that point would support the growth of *Cannabis* favorably. The Maxent software outputs a “suitability score”. Whereas species occurrences are known regions where cannabis is found and documented. The use of suitability in the historic distribution modeling section has been changed to “high potential suitability”

**Comment 15:** References are not ordered. That makes it difficult for the people to find
- There is no record in bibliography linked to Clare and Merlin, 2013: Do you mean Clarke, R. C., & Merlin?

**Response:** The references have been correctly ordered and the above reference to Clarke and Merlin, 2013 has been included.
