# Supplementary material for: Species distribution of Cannabis sativa: Past, present and future
Source: PLoS One. 2025 Mar 13;20(3):e0306007. doi: 10.1371/journal.pone.0306007 (PMC11906060; doi:10.1371/journal.pone.0306007)
Supplement: Attachment — Submitted filename: Cannabis SDM reponse to reviewer comments.docx [file pone.0306007.s005.docx]

## Editor:

Thank you for the opportunity to revise the manuscript

## Reviewer 2

**Comment:** The manuscript entitled “Species Distribution of Cannabis sativa: Past, Present and future" used species distribution modeling approach together with relevant climate variables to construct the past and predict the current and future distribution patterns of Cannabis sativa. The manuscript is well written, and the drawn conclusions are coherent with the obtained results. Although similar methodologies are common, the results of the study could have useful implications for management actions. The manuscript requires some changes before it's ready for publication.

**Response:** Thank you for the succinct summary

**Comment:** I suggest reporting the habitat areas (changes) in Kilometers for the past, current, and the future.
**Response:** For the present and day and future scenarios (both rcp and 2050/2070) we have in Table S7 the conversions to km2 worldwide as well as for the niches, as well as for specific areas of interest.

We have not done this for the past data however, as we feel that these estimations in the past for species distribution may not so easily translate accurately, given the changes in geographic landscapes in a deeper time context (rising ocean levels, glacial maximums etc). It may not be a fair estimate to compare the value in range changes in the past to the present given the occurrence of such major geological events outside of just climate properties.

**Comment:** “(12)(Zhang et al., 2018).” Please either use ‘numbered’ reference style or ‘authors, date’ style.

**Response:** Done

**Comment:** “species distribution models (SDM)” should be “species distribution models (SDMs)”
**Response:** Done

**Comment:** I suggest restructuring the methodology section into the following subsection:
1- Occurrence points
2- Environmental variables
3- Model building
4- Model evaluation (Area Under the Receiver Operating Curve (AUC)).
**Response:** Done

**Comment:** In this section, it's important to clarify what threshold was used to delineate the suitability and unsuitability areas.
**Response**: Done, as the reviewer probably accidentally overlooked this was already stated

“Suitability maps were overlaid for the present day (1970-2000), 2050 and 2070, with a suitability cutoff score of 0.2. Acceptable suitability is defined as 0.2 for cultivated regions (30) and 0.4 for natural areas (31).”

**Comment:** Implications for Future Cultivation : A small section highlighting the benefits of the applied modeling techniques in establishing priority zones for management actions is necessary. In addition, a couple of sentences on the limitations of the modeling technique is required. For this, I suggest:
[https://doi.org/10.1016/j.ecoinf.2022.101930](https://urldefense.com/v3/__https://doi.org/10.1016/j.ecoinf.2022.101930__;!!PvDODwlR4mBZyAb0!Q1SdKhRadTob3XVD_VaWHK2lsBXtEwS3RXQwKLJ3un-jwPyA4-R9Tk7p8DwecDlN_r7VdMgBXIJ5DbSseA$)
[https://doi.org/10.1007/s10661-024-12438-z
https://doi.org/10.1007/s10661-024-12438-z](https://urldefense.com/v3/__https://doi.org/10.1007/s10661-024-12438-z__;!!PvDODwlR4mBZyAb0!Q1SdKhRadTob3XVD_VaWHK2lsBXtEwS3RXQwKLJ3un-jwPyA4-R9Tk7p8DwecDlN_r7VdMgBXIIhz-q3NQ$)
[https://www.mdpi.com/2071-1050/14/21/14621](https://urldefense.com/v3/__https://www.mdpi.com/2071-1050/14/21/14621__;!!PvDODwlR4mBZyAb0!Q1SdKhRadTob3XVD_VaWHK2lsBXtEwS3RXQwKLJ3un-jwPyA4-R9Tk7p8DwecDlN_r7VdMgBXILwtJEfvQ$)

**Response:** We have added the sentences:

“Using bioclimatic and biophysical variables has shown to be effective in conservation efforts to help identify areas that should have priority for conservation (Hama and Khwarahm, 2023; Mirhashemi et al., 2024). The principal has the same potential benefit for identifying priority areas for cultivation under climate change.”

Hama, A. A., & Khwarahm, N. R. (2023). Predictive mapping of two endemic oak tree species under climate change scenarios in a semiarid region: range overlap and implications for conservation. *Ecological Informatics*, *73*, 101930.

Mirhashemi, H., Ahmadi, K., Heydari, M., Karami, O., Valkó, O., & Khwarahm, N. R. (2024). Climatic variables are more effective on the spatial distribution of oak forests than land use change across their historical range. *Environmental Monitoring and Assessment*, *196*(3), 289.

## Reviewer 3

**Comment:** This manuscript presents a novel exploration of the historical, current, and projected future distributions of Cannabis sativa. By employing species distribution modeling (SDM) based on bioclimatic and soil variables, it offers insights into how changing climate conditions could impact the range and suitability of Cannabis habitats. The modeling under paleoclimatic scenarios, coupled with present and future climate projections, adds significant value to understanding both the ecological and practical dimensions of Cannabis cultivation and conservation.

**Response:** Thank you for the summary

**Comment:** The revised manuscript addressed many initial comments, particularly in methodology justification, result interpretation, and improvements to data selection rationale. The addition of comments on data limitations, particularly regarding soil information and gaps in training data, enhances the transparency of the methodology.

**Response:** Thank you

**Comment:** I recommend that this revised manuscript be accepted for publication in PLOS ONE after addressing some remaining amendments for clarity and scientific rigor. The authors have responded well to previous comments, but additional refinements would further strengthen the manuscript.
**Response:** Thank you

**Comment:** The manuscript currently includes both numerical citations and author-based in-text citations (e.g., “Zhang et al., 2018”), which creates inconsistency. Please unify the reference style, choosing either numerical or author-date citation for all references in accordance with PLOS ONE formatting guidelines.
**Response:** Done

**Comment:** The manuscript would benefit from a clearer distinction between Cannabis species growing in the wild and those cultivated under controlled conditions. This differentiation is crucial, as cultivation practices allow for precise management of variables, whereas wild populations are subject to natural selection pressures and environmental variability. Expanding this discussion could clarify the ecological impacts and distinctions in Cannabis adaptation strategies.
**Response:** This is a contested area, what is truly wild, what is feral, and what is wild crafted. This is why we reduced the number of occurrence points to choose those that showed no indication of human intervention.

**Comment:** The authors used 137 occurrence points to model habitat suitability globally, which may be a limited representation, especially given the scale of the analysis. A discussion addressing whether this sample size sufficiently captures global suitability would be valuable, perhaps mentioning potential limitations and the implications of using Eurasian data exclusively for a global SDM.

**Response:** We have added the statement

“Further, the limited sample size of the wild occurrences may lead to increased variability.”

As for the limitation of using Eurasian samples, this was done as this is the native range for Cannabis. There are strong reasons for only using these points to explore future niches. By capturing the climate variability that the species experiences in its native range here, we can then predict suitability in other non-observed areas (such as the American continent) where climatic properties match. This therefore does not limit the potential or translation to a global SDM model when only using Eurasian data points.

**Comment:** Although the paper explores past and present distribution shifts, it lacks a focused discussion on historical changes in the distribution of Cannabis. Integrating an analysis or discussion of historical fluctuations in its range, especially in response to past climatic shifts, could provide a more comprehensive context.

**Response:** This would be quite interesting but is not the major goal of this paper. The major goal here is to explore present day potential species ranges and how potential future changes may impact cultivation areas. It is difficult to translate the range changes in the past to the effects these may have had on the evolutionary trajectory of the plant without matched DNA or pollen samples for those specific timepoints. Work by Prof John McPartland has covered an exploration of past niches with matching pollen samples and was referenced here in this publication and in discussion.

**Comment:** While the authors effectively discuss how climate variables have influenced Cannabis distribution, soil properties such as organic carbon content and pH also vary over millennia. A brief discussion on how soil characteristics have changed historically could help contextualize the model’s results, as climate and soil variability are interrelated in shaping Cannabis habitats.

**Response:** We feel that this is outside the scope of the current paper to discuss historical biogeochemical cycling in soils, as the main goal was to explore the present day and potential future ranges for the species with respect to climate. We only included the soil data for the present day data analysis as we agree, soil properties are subject to change and much more difficult to rely upon through large timeframes at such broad resolutions.

## Reviewer 4

**Comment:**The manuscript, titled "Species Distribution of Cannabis sativa: Past, Present and Future," offers valuable insights into how this species’ distribution responds to climate change. By modeling the historical, current, and projected future ranges of Cannabis sativa, the study highlights key environmental factors that shape its distribution under different climate scenarios. This research adds meaningfully to our understanding of the expansion-contraction model, especially for species affected by environmental shifts.

**Response:** thank you for the summary.

**Comment:**In terms of modeling, the use of AUC as a performance measure is helpful; however, including AICc and ROC values would make the model evaluation even more robust. AICc would allow for a clearer comparison of model fit across alternatives, while the ROC curve would provide a more complete view of predictive performance. These additions could be especially useful for readers with an interest in model selection and accuracy.

**Response:** This is a good point. We apologize but we have not included the AIC as we no longer have access to the model output and because we did not use them to make our model decisions. The ROCs for each analysis are available in the supplementary files.

**Comment:** Additionally, the discussion on expansion and contraction might benefit from referencing the study by Ülker, Tavşanoğlu, and Perktaş (2018) on Quercus robur ("Ecological niche modeling of Pedunculate Oak supports the ‘Expansion-Contraction’ model of Pleistocene biogeography"). Including this work may offer a useful comparison and add depth to the discussion of species distribution changes over time.

**Response:** we have added the sentence:

“Similar to previous work we see an expansion and contraction at known times of historical climate fluctuations (Ulker et al., 2018).”

Ülker, E. D., Tavşanoğlu, Ç., & Perktaş, U. (2018). Ecological niche modelling of pedunculate oak (Quercus robur) supports the ‘expansion–contraction’model of Pleistocene biogeography. *Biological Journal of the Linnean Society*, *123*(2), 338-347.

**Comment:** Finally, it’s clear that the authors have made considerable improvements based on previous feedback, especially in addressing the limitations of soil data, explaining data selection, and refining methodological details. These thoughtful updates have successfully resolved earlier concerns and have strengthened the manuscript overall.

**Response:** Thank you

## Reviewer 5

**Comment:** All comments have been addressed

**Response:** Thank you for looking at the specific comments we were asked to address instead of bringing up different new tangential concerns

**Comment:** In this study, authors have used various multi-source spatial and non-spatial data to investigate species Distribution of Cannabis sativa for Past, Present and future. It is an impressive compilation of information related with the documentation of Cannabis sativa.
**Response:** Thanks you for the summary

**Comment:** Authors have used CMIP5 GCM dataset for future period. Cannabis are sensitive to temperature and humidity. Previous CMIP5 models have higher uncertainty in temperature and rainfall than CMIP6 GCMs. I suggest applying a dataset of CMIP6 GCMs, otherwise provide uncertainty in estimated suitability areas due to the use of CMIP5 GCMs.

**Response:** We apologize if this was unclear, but this is incorrect, we stated in the methods we used CMIP6
